# Primary oesophageal small cell carcinoma initially manifestating as purulent pericardiac effusion

**DOI:** 10.1259/bjrcr.20160051

**Published:** 2016-09-22

**Authors:** Go Eun Yang, Heung Cheol Kim, Jung Hun Kim

**Affiliations:** ^1^Department of Radiology, Hallym University College of Medicine, Chuncheon, South Korea; ^2^Department of Oncology, Hallym University College of Medicine, Chuncheon, South Korea

## Abstract

This article reports a case of primary oesophageal small cell carcinoma with a large amount of purulent pericardial effusion due to oesophageal rupture. The patient was treated with chemotherapy, which resulted in a marked decrease in the size of the oesophageal mass and partial resolving of metastatic lymphadenopathy.

## Introduction

Primary oesophageal small cell carcinoma (POSC) is a rare malignant tumour that was first reported by McKeown in 1952. This disease has the characteristics of high malignancy, local recurrence, high metastatic rate and short survival time.^1^

Four cases of oesophageal–pericardial fistula with purulent pericarditis secondary to oesophageal carcinoma have been previously reported.^2^ In two cases, cardiac tamponade was the first manifestation of oesophageal carcinoma. Histopathologically, most of these cases were squamous cell carcinoma or unknown.

We recently encountered a 44-year-old male with POSC manifesting as purulent pericarditis.

## Case report

A 44-year-old male was admitted with dyspnoea that had progressively worsened 3 days earlier. He complained of recent chest pain in the midsternum, and left arm oedema and weight loss for 4 months. Vital signs at presentation were blood pressure of 100/80 mmHg, pulse of 98 beats min^−1^, temperature of 36.6°C and oxygen saturation of 100%. There was no known underlying disease.

**Figure 1.**
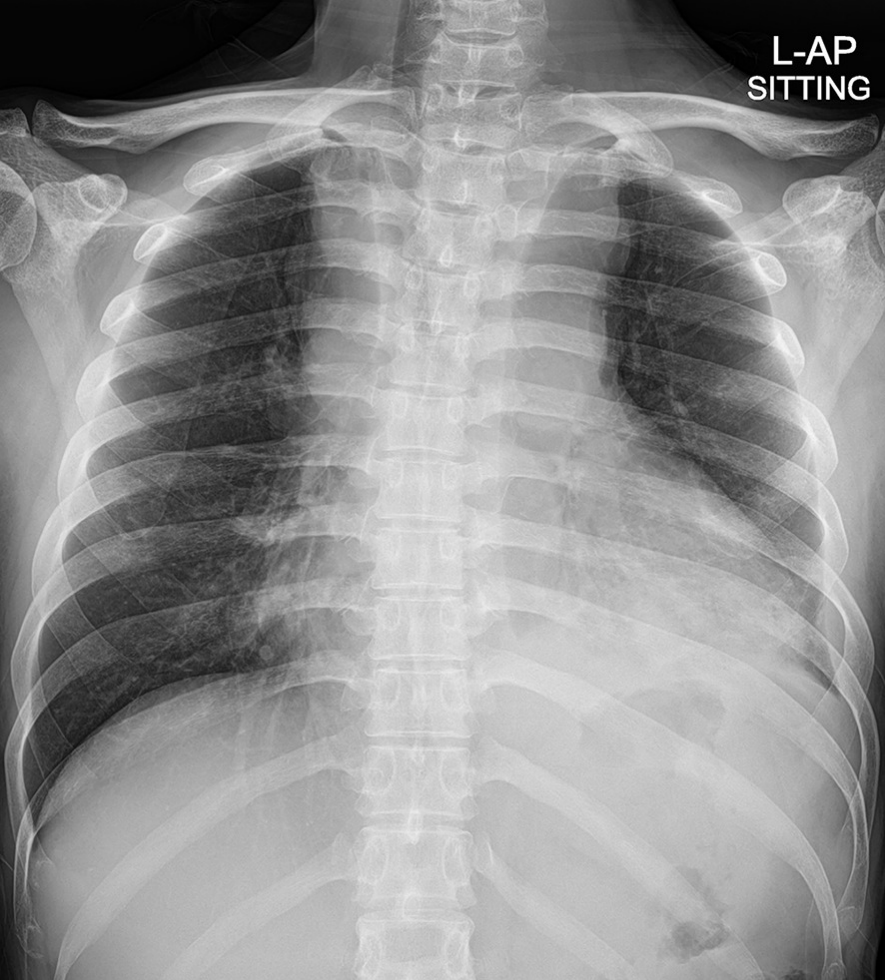
Initial chest AP radiograph showing mediastinal widening and nodular consolidation in the left lower lobe and pleural effusion. The trachea is deviated to the right side. L-AP, left anteroposterior.

A chest radiograph showed mediastinal widening, nodular consolidation in the left lower lobe and pleural effusion (Figure 1).

Blood urea nitrogen/creatinine level was elevated at the time of analysis (79.9/4.7), so a CT scan was performed without contrast enhancement.

**Figure 2.**
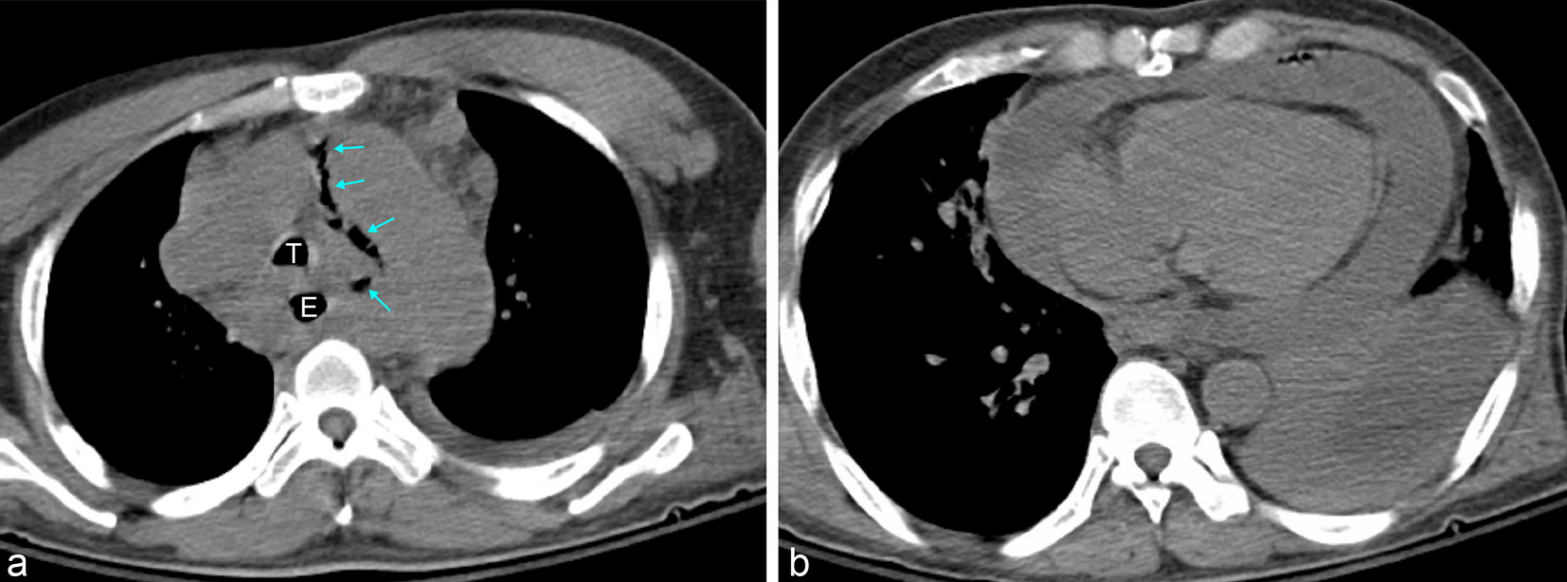
(a) A CT scan showing a huge lobulated mass involving the oesophagus (E), mediastinum and lower neck (not revealed) with enlarged lymph nodes. The scan reveals a large amount of pericardial effusion and extraluminal air bubbles at the mediastinum (arrows). A relatively preserved trachea (T) is noted without definite extrinsic compression. (b) A large amount of pericardial effusion and extraluminal air bubbles along the pericardium are seen. Consolidation in the left lower lobe with left pleural effusion is noted.

The CT scan showed a huge lobulated mass involving the oesophagus, mediastinum and lower neck with enlarged lymph nodes (Figure 2a,b).

The analysis revealed a large amount of pericardial effusion and extraluminal air bubbles along the pericardium, suggesting pneumomediastinum (Figure 2a) and pneumopericardium (Figure 2b).

Echocardiography and pericardiocentesis were performed, resulting in the drainage of 300 ml of pus-like light brown fluid.

**Figure 3.**
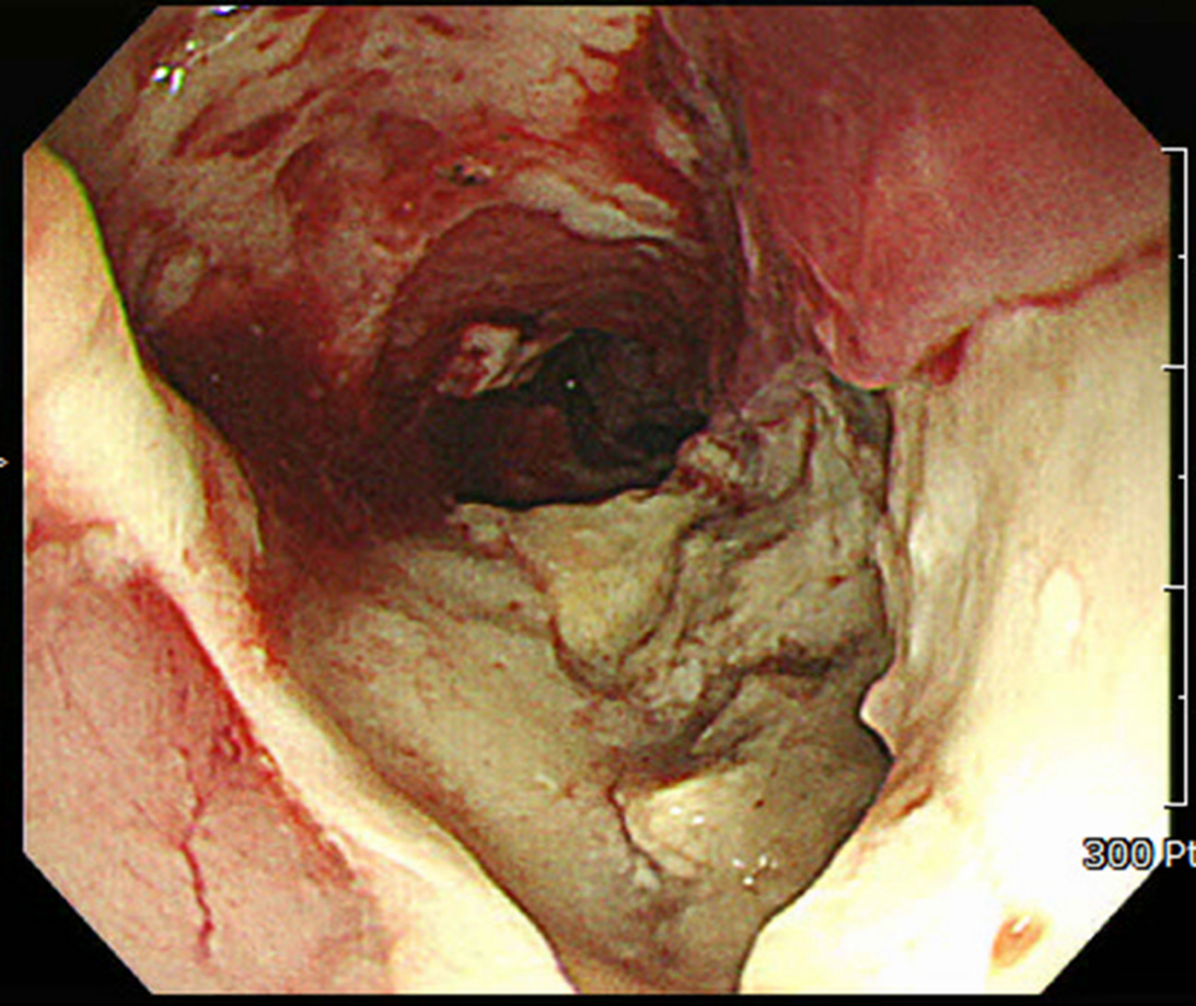
Endoscopic study revealing a huge oesophageal ulcer encircling the oesophageal lumen at 20–30 cm from the incision.

An endoscopic study revealed a huge oesophageal ulcer encircling the oesophageal lumen at 20–30 cm from the incision (Figure 3).

The stomach showed multiple erythematous plaques and erosions, and mild atrophic changes of the mucosa but no definite mass lesion.

**Figure 4.**
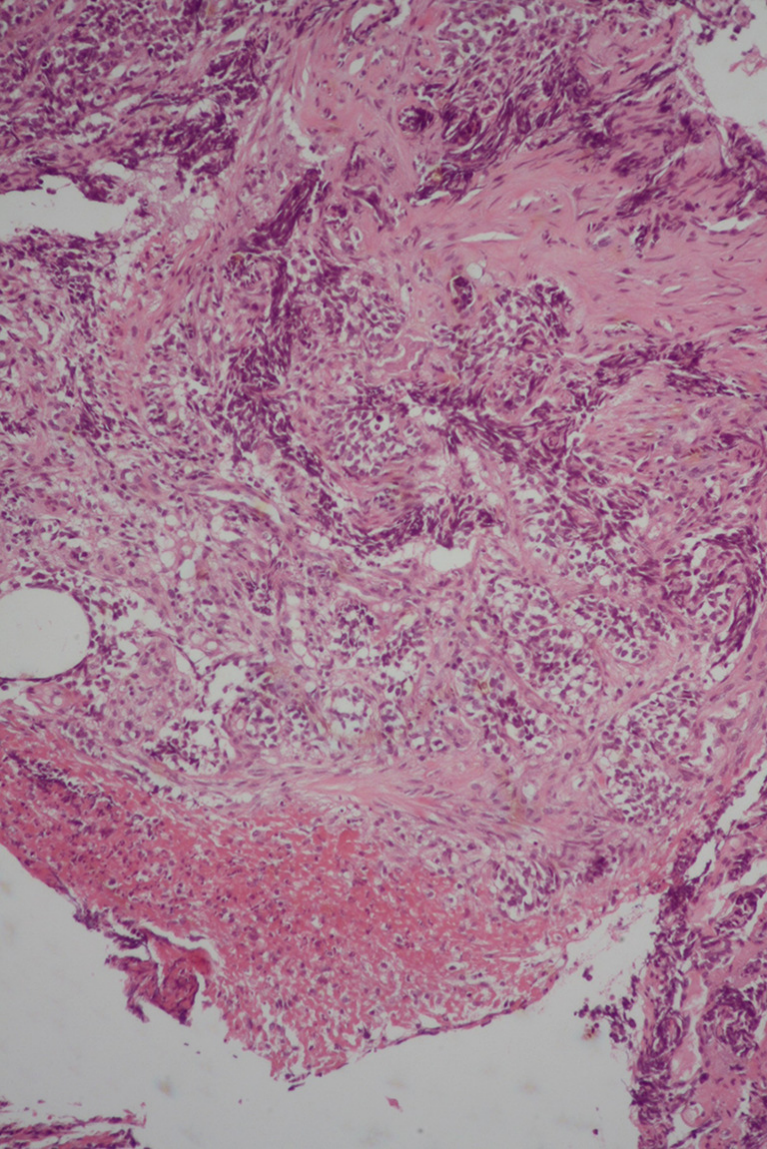
Lower magnification view of an oesophageal biopsy specimen shows infiltrating tumour tissue arranged in solid nests. Tumour necrosis is also noted (haematoxylin and eosin, ×100).

**Figure 5.**
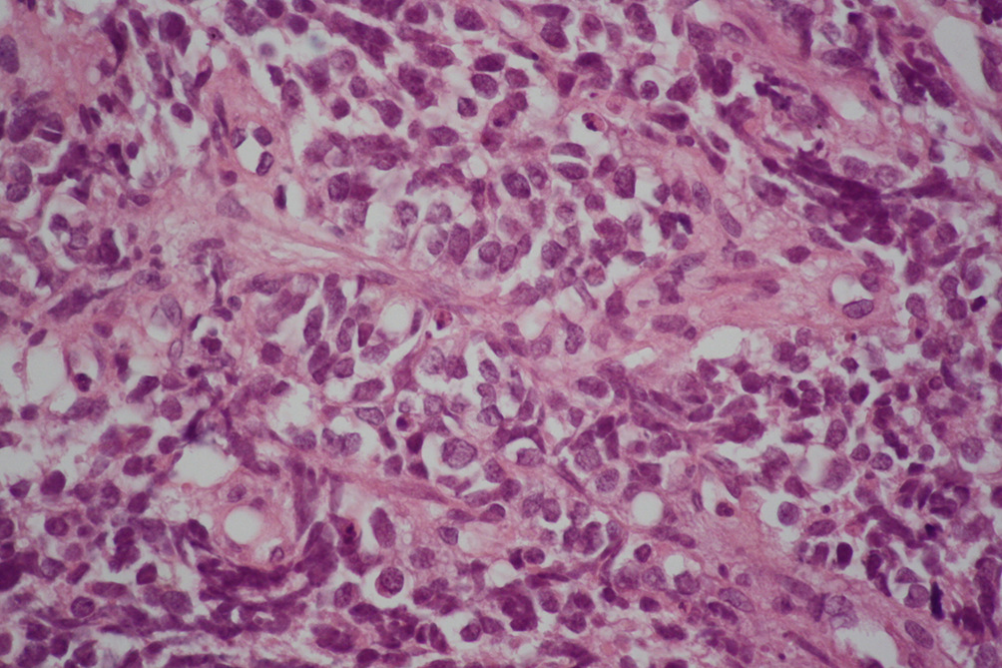
Higher magnification view of an oesophageal specimen shows small hyperchromatic cells with finely granular chromatin, inconspicuous nucleoli, scanty cytoplasm and nuclear moulding (haematoxylin and eosin, ×400).

**Figure 6.**
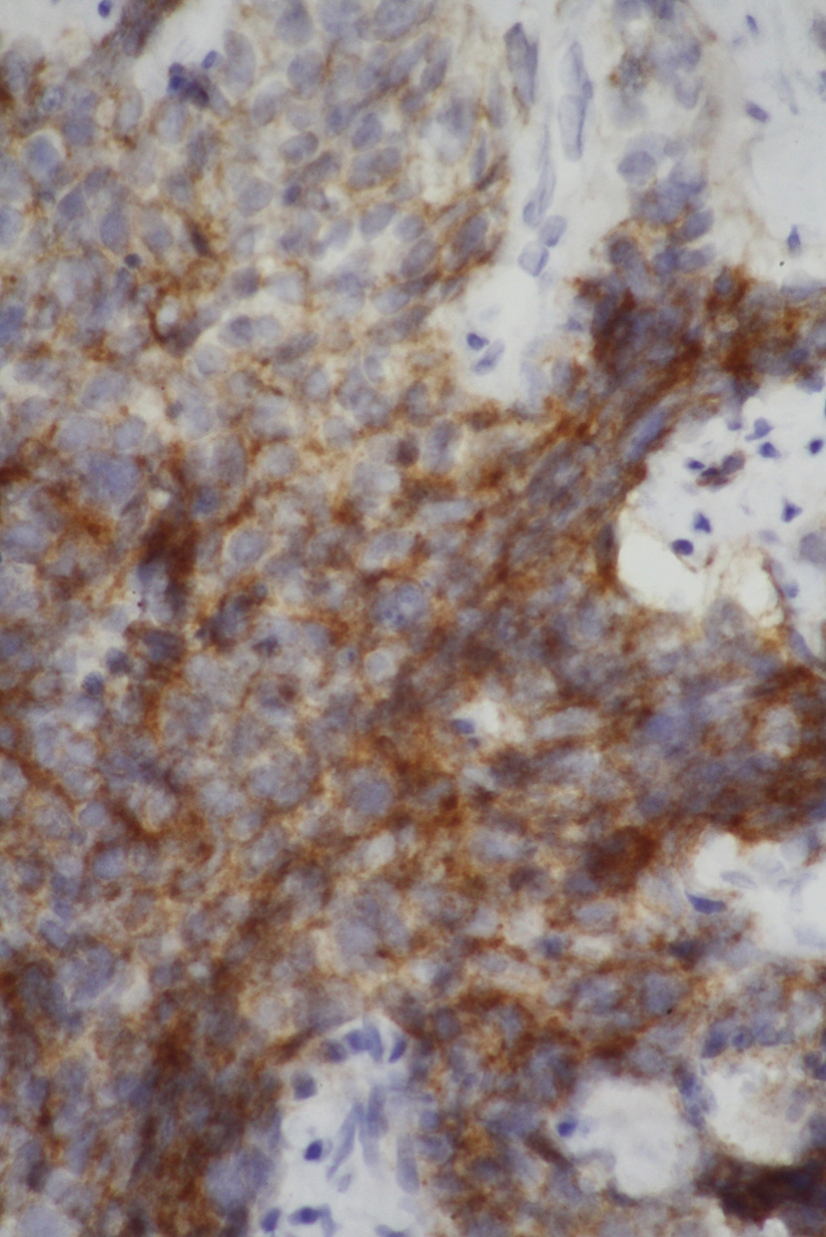
Tumour cells stained positive for CD56 (×400).

**Figure 7.**
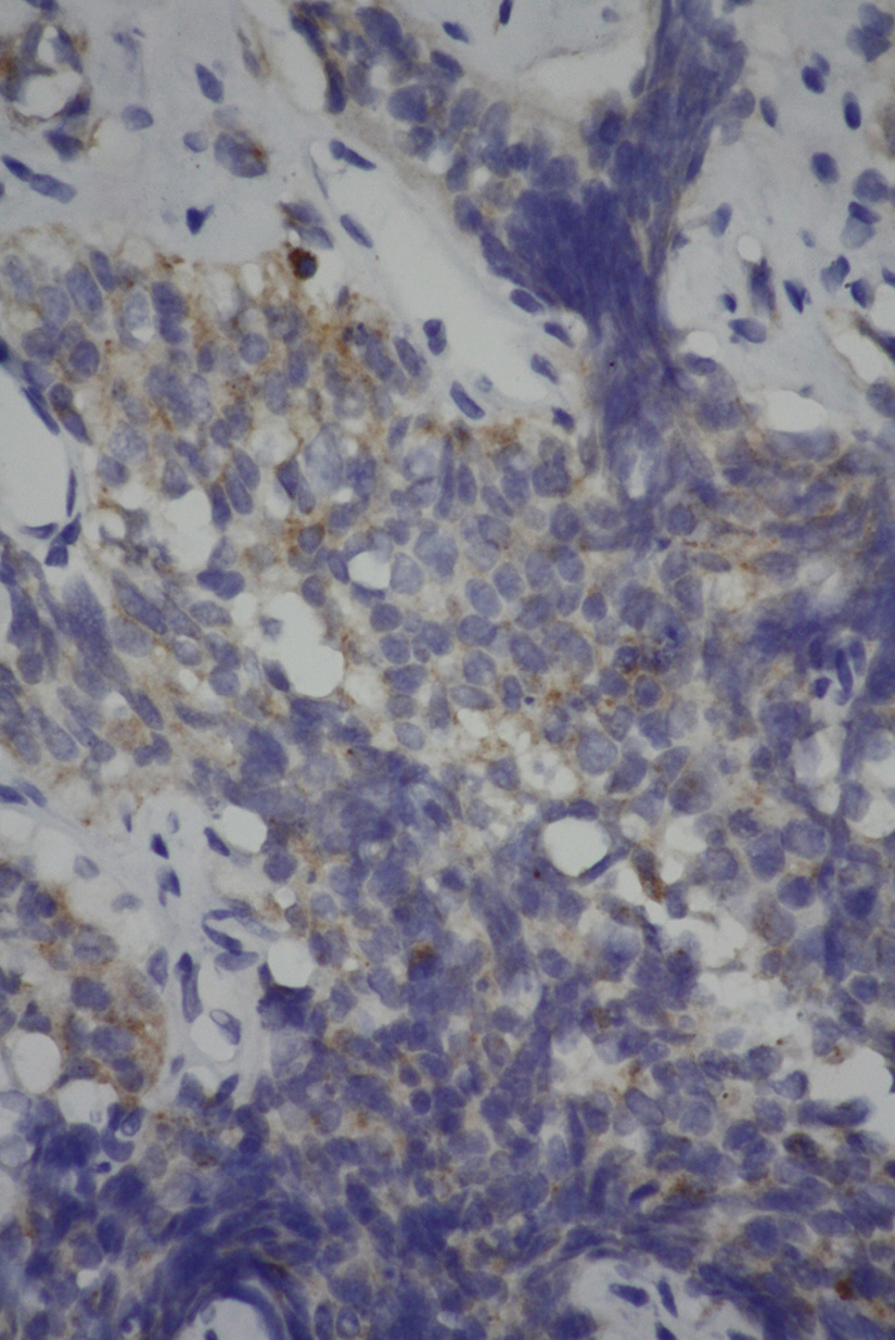
Tumour cells stained positive for synaptophysin (×400).

Endoscopic biopsy was performed of the oesophageal lesion and histological analysis of the biopsies suggested small cell carcinoma (SCC; Figures 4–7). On immunohistochemistry, the tumour cells stained positive for CD56 and synaptophysin (endocrine markers) and were negative for p63 and leukocyte common antigen, excluding squamous cell carcinoma and lymphoma.

**Figure 8.**
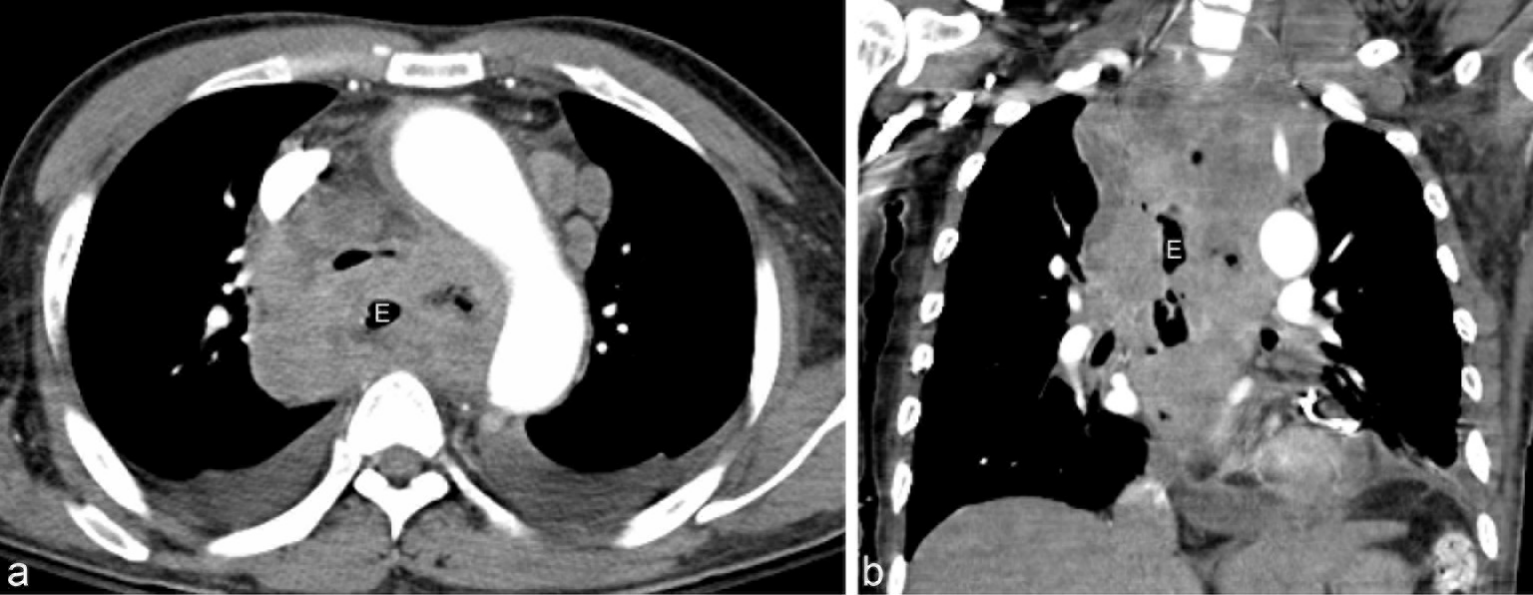
Chest axial (a) and coronal reconstructed (b) CT with contrast enhancement was performed. Residual pericardial effusion and thin diffuse pericardial enhancement were seen in addition to a huge mass encircling the oesophagus, mediastinum and lower neck, and metastatic lymphadenopathy.

An enhanced CT scan study performed 4 days after presentation (Figure 8) showed residual pericardial effusion with thin diffuse pericardial enhancement and a huge mass encircling the oesophagus, mediastinum and lower neck with metastatic lymphadenopathy.

Small nodules in the left lower lobe with pleural effusion suggested pneumonia or metastasis to the lung.

**Figure 9.**
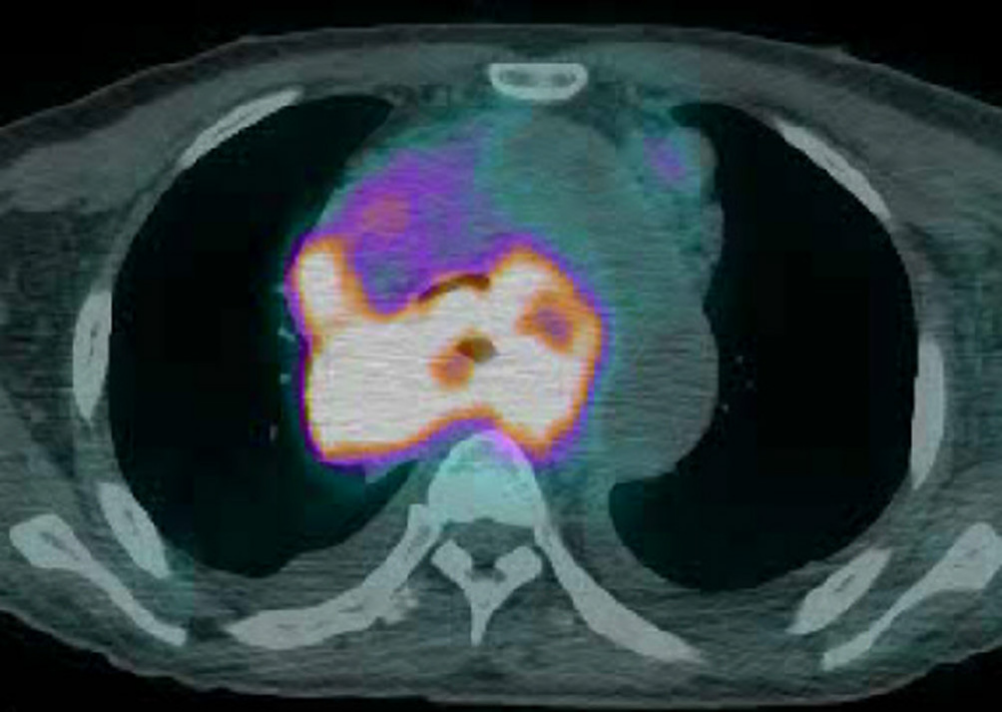
Positron emission tomography/CT scan was performed on the same day with enhanced CT scan, showing very strong uptake at the mediastinal mass and lymph nodes.

Positron emission tomography/CT scan was performed on the same day, and very high uptake was noted by the mediastinal mass and lymph nodes (Figure 9).

**Figure 10.**
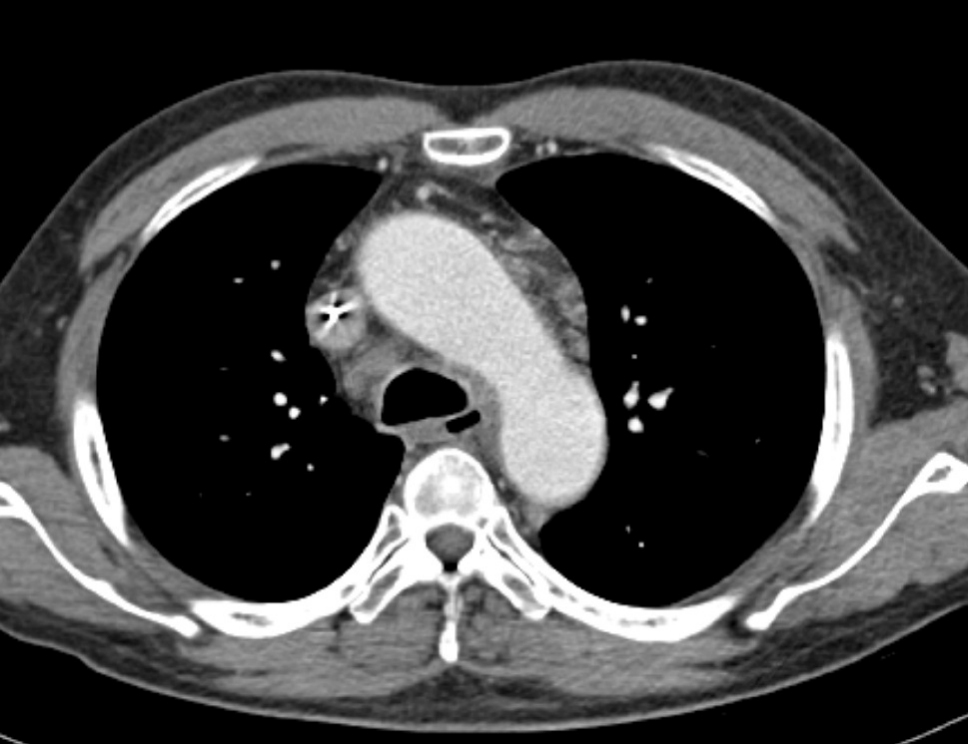
Chest CT scan with contrast enhancement was performed after 5 months from the initial CT scanning (after seven cycles of chemotherapy). The image shows markedly decreased size of the mediastinal mass, leading to improvement in dyspnoea, and left arm oedema. Residual pericardiac effusion and thin diffuse pericardial enhancement were seen in addition to a huge mass encircling the oesophagus, mediastinum and lower neck, and metastatic lymphadenopathy.

The patient was treated with chemotherapy with cisplatin and etoposide, which markedly decreased the size of the mediastinal mass and improved the dyspnoea and left arm oedema (Figure 10).

Antibiotic treatment was administered and improved the pneumonia in the left lower lung.

After eight cycles of chemotherapy, the masses showed partial response; however, multiple brain metastases developed 7 months after the initial diagnosis. A poor prognosis is expected.

## Discussion

About 5% of cases of SCC have an extrapulmonary origin. Primary SCC of the oesophagus was first described in 1952 and has been reported to have a variable incidence rate of 0.5–2.4% in different series.^3^ Similar to pulmonary SCC, SCC of the oesophagus is an aggressive tumour associated with a poor prognosis.^^3–5^^

Many cases of primary SCC of the oesophagus have been reported.^^1,3–10^^ Four cases of oesophageal–pericardial fistula with purulent pericarditis secondary to oesophageal carcinoma have been reported in the literature. In two of these cases, cardiac tamponade was the first manifestation of carcinoma of the oesophagus.^2^

- Histopathologically, most of these cases were squamous cell carcinoma or unknown. The patients were treated with pericardial drainage and subsequent oesophageal stenting.^11^
- Here, we report a case of POSC manifesting as purulent pericardial effusion, suggesting oesophageal–pericardial fistula.
- In conclusion, POSC is an extremely rare malignancy and represents up to 0.05–4% of all oesophageal malignancies. Patients with POSC usually experience short survival, often with early distant metastasis.^6^ Thus, while oesophageal carcinoma with fistula is very rare, it should be included in the differential diagnosis of purulent pericardial effusion. In cases of SCC, chemotherapy can be the adjuvant treatment.

## Learning points

1. Primary oesophageal carcinoma with purulent pericarditis also can result in cardiac tamponade, which is a life-threatening condition.
2. Oesophageal–pericardial fistula with purulent pericarditis secondary to oesophageal carcinoma is an important manifestation of carcinoma of the oesophagus.
3. POSC is a rare, highly aggressive tumour associated with a poor prognosis, similar to SCC of the lung or other extrapulmonary organs.
4. Primary oesophageal SCC can be treated with the same type of chemotherapy as used for treating pulmonary or extrapulmonary SCC.

## Consent

The patient signed written informed consent for the case to be published.
